# Path model explaining the association between fear of falling and health-related quality of life in (pre-)frail older adults

**DOI:** 10.1186/s12877-025-05718-x

**Published:** 2025-02-07

**Authors:** Tjard Sattler, Sophie Gottschalk, Hans-Helmut König, Tobias Braun, Gisela Büchele, Michael Denkinger, Tim Fleiner, Corinna Nerz, Kilian Rapp, Martina Schäufele, Christian Werner, Judith Dams

**Affiliations:** 1 Department of Health Economics and Health Services Research, University Medical Center Hamburg-Eppendorf, Martinistraße 52, 20251 Hamburg, Germany; 2 Hamburg Center for Health Economics, Hamburg, Germany; 3 Department of Applied Health Sciences, Hochschule für Gesundheit, Bochum, Germany; 4 Department of Health, HSD Hochschule Döpfer, Cologne, Germany; 5 Institute of Epidemiology and Medical Biometry, Ulm University, Ulm, Germany; 6 Institute for Geriatric Research, Ulm University Medical Center, Ulm, Germany; 7 Institute of Medical Engineering and Mechatronics, Ulm University of Applied Sciences, Ulm, Germany; 8 Department of Clinical Gerontology, Robert-Bosch-Hospital, Stuttgart, Germany; 9 Department of Social Work, Mannheim University of Applied Sciences, Mannheim, Germany; 10 Geriatric Centre, Heidelberg University Hospital, Agaplesion Bethanien Hospital Heidelberg, Heidelberg, Germany

**Keywords:** Fear of falling, Physical capacity, Physical performance, Health-related quality of life, Path analysis, Frailty

## Abstract

**Background:**

Fear of falling (FoF) is estimated to be prevalent in over 50% of older adults and several studies suggest that it negatively affects health-related quality of life (HrQoL). Unlike previous studies that examined only few mediating variables, this study aimed to develop a more comprehensive path model explaining the association between FoF and HrQoL.

**Methods:**

A theoretical path model was developed based on existing evidence and expert feedback and fitted to cross-sectional baseline data on 385 community-dwelling (pre-)frail older adults from the PromeTheus randomized controlled trial using robust weighted least squares estimation. FoF and HrQoL were operationalized by the Short Falls Efficacy Scale International and EQ-5D Index, respectively. The model included potential explanatory pathways through physical activity (German Physical Activity Questionnaire for middle-aged and older adults), physical capacity (Short Physical Performance Battery), physical performance (Late-Life Function and Disability Instrument [LLFDI] function component), disability (LLFDI disability component – short form), and affect (visual analogue scales on ‘happiness’, ‘sadness’, ‘calmness’ and ‘tension’). Age, sex, education, and previous falls were considered as covariates.

**Results:**

The model demonstrated good fit to the data and the remaining direct effect of FoF on HrQoL was small (β=-0.05). Physical capacity and physical performance were the most important mediators (combined indirect effect of β=-0.17, accounting for > 50% of the total effect). Pathways of minor individual relevance (e.g. through disability or affect) contributed considerably to the total indirect effect when combined. Controlling for sociodemographic data and previous falls only had minor effects on model fit and path coefficients.

**Conclusion:**

Physical capacity and physical performance are particularly important levers for reducing the impact of FoF on HrQoL through interventions. However, the other pathways also had a considerable influence when taken together. Hence, research on the association of FoF and HrQol should acknowledge the complexity of causal pathways that may explain this association and not neglect minor pathways. The proposed model should be tested on an alternative sample, using longitudinal data, and extended to include additional explanatory factors (e.g. activity avoidance).

**Trial registration:**

German Clinical Trials Register, ID: DRKS00024638, https://drks.de/search/en/trial/DRKS00024638, date of registration: March 11th 2021.

**Supplementary Information:**

The online version contains supplementary material available at 10.1186/s12877-025-05718-x.

## Background

Falls are a prevalent health issue in older adults, having a relevant impact on the burden of disease and consequently on quality of life (QoL) in this population [1, 2]. Therefore, research into effective fall prevention has become a growing field [3, 4]. Fear of falling (FoF) is a psychological aspect of falling that can be described as “low perceived self-efficacy at avoiding falls during essential, nonhazardous activities of daily living” [5]. Prevalence estimates vary, but a recent meta-analysis estimated FoF being prevalent in almost half of the population aged 60 years and older [6]. In (pre-)frail older populations, the prevalence may even be as high as 75% [7, 8]. Frailty describes a state of reduced physiological reserves caused by declines in various systems, leading to an increased vulnerability to stressors. Persons who show only some elements of frailty (e.g. shrinking, weakness, poor endurance and energy, slowness, and low physical activity level) are considered pre-frail [9].

The (risk) factors associated with FoF include demographic characteristics (e.g., female gender), physical function, chronic diseases, and mental problems, while previous fall experience tends to play a minor role [6, 10, 11]. Several studies suggest that FoF is associated with lower (health-related) QoL (HrQoL) and this association also appears to be largely independent of whether a person has actually experienced a fall [12, 13]. (Hr)QoL is a key indicator for active aging and an important outcome in studies examining interventions aiming to promote active aging [14]. Understanding the mechanisms underlying the association between FoF and (Hr)QoL may help in designing effective strategies to address FoF and increase (Hr)QoL.

Just as FoF is not necessarily a consequence of previous fall experiences, perceived and physiological fall risk do not always appear to be congruent [15]. However, FoF may lead to changes in behavior such as fear-related activity restriction that cause gait speed adaptions, which (in the long term) potentially results in lower physical capacity and performance [16–19] and, in turn, increases the physiological fall risk and further intensifies FoF. These associations of FoF with physical capacity or physical performance (via activity restrictions/avoidance) present a potential linking factor in the association between FoF and (Hr)QoL. This is supported by previous studies that found, e.g., subjective functional capacity, gait speed, lower leg strength, or physical activity to partly explain the association of FoF and HrQoL (partial mediation) [20–22]. These studies used relatively simple path models that examined only one or two potential mediators at a time, making it difficult to estimate the relative importance of different explanatory factors. However, path analysis is capable of describing complex relations between various variables, allowing the evaluation of hypothesized models [23]. Compared to the existing literature, the current study aimed to include more parameters, such as physical capacity and affect, and thus provide a more detailed insight into the association between FoF and (Hr)QoL.

Therefore, the present study aimed to explain the relationship between FoF and HrQoL by including several factors known to be associated with FoF and/or HrQoL in a path model, making it possible to compare their importance and examine their interplay and dependencies.

## Methods

### Study design and sample

This study is a secondary, cross-sectional analysis using data from the baseline examination of the PromeTheus multicenter randomized-controlled trial (registered in the German Clinical Trials Register on March 11, 2021; ID: DRKS00024638) [24]. The study population consisted of (pre-)frail older adults (Clinical Frailty Scale [25] score 4–6) of at least 70 years who were living at home or in assisted living facilities in the areas of Stuttgart, Heidelberg, and Ulm (Baden-Wuerttemberg, Germany), were insured with the ‘Allgemeine Ortskrankenkasse (AOK) Baden-Württemberg’ (a German statutory health insurance), and were able to walk at least 10 m with or without walking aids but less than 800 m without walking aids and breaks. Eligibility criteria are described in detail elsewhere [24].

### Hypothesized model

**Fig. 1:**
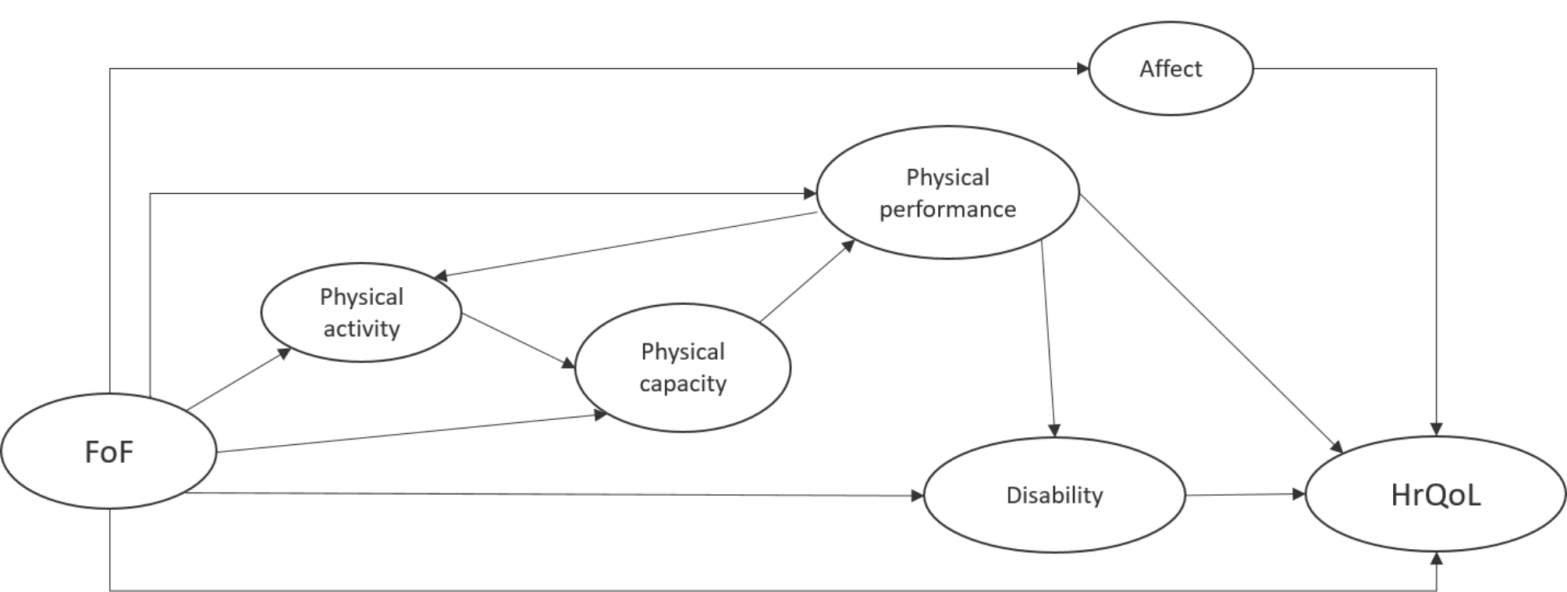
Hypothesized path model

In a first step, two authors conducted a literature review on the relationship between FoF and HrQoL. Based on the quantitative and qualitative evidence identified, they hypothesized a first path model linking FoF and HrQoL through constructs that could be measured using data from the PromeTheus study. This was modified following discussion with a group of experts (physiotherapists/sports scientists/geriatric researchers from the PromeTheus study group), so that further instruments capturing the abstract concepts were identified. Inclusion of the expert group’s feedback on this updated model led to the hypothetical path model used for this analysis (Fig. 1).

Given the proposed association between FoF and HrQoL [12] and to be able to differentiate between the direct and indirect association between FoF & HrQoL, a direct path was drawn from FoF to HrQoL. We hypothesized that a key explanatory pathway is through *mobility* (defined as the ability to move [26]), which is a determinant of older people’s HrQoL [27]. We follow the recommendation to differentiate two constructs of mobility: physical capacity (the capability or the ‘can do’ measured under standardized/ideal conditions) and physical performance (measured embedded within a (daily) task/activity and representing the ‘do’) [26]. Previous research suggests that FoF leads to avoidance or restriction of activities [16]. As this was not directly measured in PromeTheus, we assumed that these activity restrictions would present as *changes in physical capacity measures*. These could be a direct (and possibly conscious) manifestation of fear-related avoidance behavior (e.g., reduction of gait speed) or a physiological consequence of fear-related avoidance and thus non-use, which manifests itself in an actual reduction in physical capacity [18, 19, 22]. Activity avoidance may also be reflected in FoF-related reduction in *physical activity* level [20], which is a determinant of maintaining physical capacity and physical performance [28]. On the one hand, physical capacity logically affects *physical performance*; on the other hand, FoF could have a direct negative influence on physical performance regardless of capacity limitations (i.e. a person who essentially ‘can do’ certain activities might not actually ‘do’ them in daily tasks/activities). Not doing daily tasks/activities (i.e. limited in physical performance) could lead to a reduction of physical activity, which in turn could start the vicious cycle of (further) decreasing physical capacity and performance. FoF-related limitations in physical performance may, depending on a person’s surroundings and adaptability, carry over into *disability*, i.e. limited performance of socially defined life tasks [26], but FoF could also result in a person experiencing disability without being limited in physical performance per se (e.g. through avoidance). Finally, a pathway was drawn connecting FoF and HrQoL through *affect*, assuming that FoF as a psychological construct might impact the affective state (feelings, mood) more globally [29], which in turn might be reflected in HrQoL [30].

### Measures

Fear of Falling was assessed using the Falls-Efficacy Scale International – Short Form (Short FES-I), a seven-item questionnaire on concern about falling [31]. A total score was calculated from seven items regarding the concern about falling in executing everyday tasks, each item having response options from 1 (not at all concerned) to 4 (very concerned). Thus, the total score ranges from 7 (no concern about falling) to 28 (severe concern about falling). Measurement properties of the Short FES-I were sufficient, showing good test-retest-reliability (*r* = .87), very good score reliability (Cronbach’s alpha 0.92), and a strong correlation with the original and cross-culturally validated 16-item FES-I (*r* = .97) [31, 32].

HrQoL was determined by the EQ-5D-5 L index score [33]. It summarizes five dimensions of HrQoL (mobility, self-care, usual activities, pain/discomfort, anxiety/depression) into a score using health state preferences of the German general population (0 representing ‘death’, 1 representing ‘full health’, and negative scores indicating health states valued worse than death). The EQ-5D-5 L showed sufficient construct validity in older populations [34].

Physical capacity was measured using the Short Physical Performance Battery (SPPB), which assesses lower extremity function based on the three subtests: a hierarchical standing balance test (Romberg, semi-tandem, and tandem stance), a usual gait speed test over 4 m, and a 5-chair stand test [35]. A total score ranging from 0 (worst) to 12 (best) was calculated. The SPPB demonstrated good validity and reliability in frail older adults without severe cognitive impairment [36].

Physical performance was operationalized using the Late-Life Function and Disability Instrument’s (LLFDI) function component, consisting of 32 items assessing limitations in a person’s ability to perform discrete actions/activities encountered in daily routines [37]. A scaled score was calculated ranging from 0 to 100, with higher scores indicating better performance. The measurement properties of the LLFDI function component are supported by several studies [38].

Self-reported physical activity was measured using the German Physical Activity Questionnaire for middle-aged and older adults (German PAQ 50+) [39]. Participants were asked how much time they spent on a number of activities in a typical week of the last month. These times were multiplied with the metabolic equivalent (MET) for the respective activity [40] and summed to calculate the activity level as MET-hours per week. The instrument was constructed from other validated instruments, indicating good construct validity; test-retest-reliability was insufficient (*r* = .53) [39].

Disability – the ability to perform socially defined life tasks within a typical sociocultural and physical environment [41] – was assessed with the short form of the LLFDI disability component’s limitation dimension [42, 43]. The raw score ranging from 8 to 40 was calculated, with higher scores indicating a lower level of disability. It has been found to have sufficient reliability and validity [43, 44].

Affect was measured on visual analogue scales to four questions regarding ‘happiness’, ‘sadness’, ‘calmness’ and ‘tension’, which were summarized to a score between 0 (high level) and 100 (low level of affect) [45]. Even though the instrument was designed to detect individual changes over time, its known-groups validity provides some evidence for its use in inter-individual comparison [45].

Furthermore, self-reported information on age, gender (male/female), years of formal education, and whether participants had fallen in the last six months (yes/no) were used as control variables.

### Statistical analysis

All analyses were performed using R version 4.2.3 software. Data was complete for the variables of interest for this analysis, except for occasional missing values in the variables ‘affect’ (*n* = 1) and ‘years of education’ (*n* = 2), which were replaced by the median of the respective variable.

Descriptive and bivariate statistics were used to describe the sample characteristics and test statistical requirements for subsequent analyses.

The lavaan package (version 0.6–16) for R was used for path analysis [46]. In path analysis, simultaneous regression analyses are conducted between certain variables according to a pre-specified model, allowing the estimation of both direct and indirect effects. The theoretical model was fitted using robust weighted least squares estimation which does not assume normality of the data used and allows analysis of both metric and dichotomous variables [47]. Some variables – namely physical activity, physical performance, disability, HrQoL, education, sex, and previous falls – were multiplied with a constant factor to ensure model convergence. Modification indices were inspected to identify potential additional connections between variables that would improve the model’s fit. Removing regressions with minimal effect from the model was considered in the interest of parsimony, but was ultimately rejected because of the theoretical or practical significance of the affected pathways. The model was analyzed both in a raw version and in one that controlled for age, sex, education, and previous falls.

Following the recommendations by Kline [47], global fit of the model was examined by Chi-squared statistics, the robust Root Mean Square Error of Approximation (RMSEA), and the Standardized Root Mean Square Residual (SRMR) as absolute fit measures, as well as the robust Comparative Fit Index (CFI) as measure for incremental fit. A non-significant Chi-squared statistic on a 0.05 confidence level, a CFI ≥ 0.90, RMSEA < 0.05, and SRMR values < 0.08 were considered indicative for good model fit [48]. Correlation residuals < 0.10 were taken as indicators of good local fit [47]. Standardized and unstandardized path coefficients as well as indirect effects were calculated alongside 95%-confidence intervals (CIs).

## Results

### Descriptives

Sample characteristics of the 385 baseline participants of PromeTheus are presented in Table 1. The mean age was 81.2 years, the majority was female (73.5%) and either married (30.6%) or widowed (51.7%). 32.2% were living in an assisted living facility, 38.5% had a care degree (qualifying for benefits from the German long-term care insurance), and 36.9% reported at least one fall in the last 6 months. Their median score on the clinical frailty scale was 4 (interquartile range 4–5), indicating very mild to mild frailty. The mean and standard deviation of variables of interest in the path model (HrQoL, FoF, physical activity, physical capacity, physical performance, disability, and affect) as well as their bivariate correlations (Spearman’s rank correlation coefficient, ρ) are provided in Table 2. There was a moderate negative bivariate correlation between FoF and HrQoL (ρ=-0.35). Further, HrQoL and FoF correlated moderately to strongly (ρ ≥ 0.3) with physical capacity, physical performance, and disability, while the associations with physical activity and affect were weaker.

**Table 1:** Sample characteristics (*n* = 385)

Age - mean (SD)	81.2 (5.9)
Female - n (%)	283 (73.5)
Family status - n (%)	
Married	118 (30.6)
Married, living separated	4 (1.0)
Single	26 (6.8)
Divorced	38 (9.9)
Widowed	199 (51.7)
Years of education – mean (SD)	11.29 (2.8)
Living situation - n (%)	
Private household	261 (67.8)
Assisted living	124 (32.2)
Care degree^a^ (range: none and 1 to 5) - n (%)	
None	236 (61.5)
Level 1	53 (13.8)
Level 2	78 (20.3)
Level 3	17 (4.4)
Use of an assistive medical device - n (%)	273 (70.9)
Body mass index - mean (SD)	29.4 (5.8)
Clinical frailty scale (range: 1 to 9) - median (IQR)	4 (4, 5)
At least one fall within the last 6 months – n (%)	142 (36.9)
SPPB score (range: 0 to 12) – median (IQR)	6 (5, 8)

^a^German „Pflegegrad“

**Table 2:** Correlation coefficients (spearman) and means (standard deviations) of main variables included in the model

	HrQoL	FoF	Activity	Capacity	Performance	Disability	Affect
HrQoL	1						
FoF	− 0.348*	1					
Activity	0.201*	− 0.180*	1				
Capacity	0.353*	− 0.507*	0.306*	1			
Performance	0.489*	− 0.634*	0.281*	0.731*	1		
Disability	0.406*	− 0.478*	0.264*	0.514*	0.720*	1	
Affect	0.281*	− 0.225*	0.127	0.180*	0.253*	0.325*	1
Mean (SD)	0.74 (0.22)	12.56 (4.38)	69.58 (41.48)	6.46 (2.67)	47.58 (7.71)	29.52 (6.97)	67.63 (20.13)

Notes: Activity = physical activity; Capacity = physical capacity; FoF = fear of falling; HrQoL = health-related quality of life; Performance = physical performance; SD = standard deviation. Correlations which are significant on a 0.05 level are marked with an *.

### Path model

**Fig. 2:**
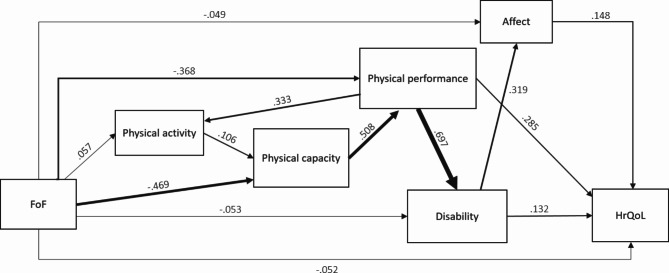
Final path model. Standardized path coefficients are shown

During model fitting, indicated by a high modification index, the hypothetical model was extended by adding a path from disability to affect, which has some theoretical support [49]. Model fit statistics indicated a satisfactory global (Table 3) and local fit (data not shown) of the resulting model in both its raw and corrected form. Fitted covariance matrices can be found in the Supplemental material (Tables S1-S2) along with the coefficients of the raw model (Table S3); coefficients of the final corrected model are presented in Table 4; Fig. 2. Overall, correcting for age, sex, education, and previous falls only slightly changed the path coefficients compared to the raw model.

**Table 3:** Model fit indices

Model	χ2 (df), *p*-value	χ2 scaled (df), *p*-value	CFI	RMSEA (95% CI)	SRMR
Raw	2.493 (7), *p* = .928	6.766 (7), *p* = .454	1.000	0.000 (0.000, 0.041)	0.017
Corrected	1.651 (7), *p* = .977	5.391 (7), *p* = .612	1.000	0.000 (0.000, 0.033)	0.009

**Table 4:** Path coefficients of the corrected model

	Path coefficient	95% CI
**Direct effects**		
HrQoL ~ FoF	−0.052 (–0.027)	(–0.193, 0.088)
HrQoL ~ Performance	0.285 (0.403)	(0.086, 0.484)
HrQoL ~ Disability	0.132 (0.208)	(–0.040, 0.304)
HrQoL ~ Affect	0.148 (0.016)	(0.040, 0.256)
Disability ~ FoF	−0.053 (–0.018)	(–0.158, 0.052)
Disability ~ Performance	0.697 (0.634)	(0.602, 0.792)
Affect ~ FoF	−0.049 (–0.224)	(–0.168, 0.070)
Affect ~ Disability	0.319 (4.636)	(0.189, 0.449)
Performance ~ FoF	−0.368 (–0.126)	(–0.444, −0.292)
Performance ~ Capacity	0.508 (0.292)	(0.424, 0.591)
Capacity ~ FoF	−0.469 (–0.286)	(–0.544, −0.394)
Capacity ~ Activity	0.106 (0.068)	(0.007, 0.204)
Activity ~ FoF	0.057 (0.046)	(–0.089, 0.203)
Activity ~ Performance	0.333 (0.873)	(0.161, 0.506)
**Selected indirect effects/paths**		
Total indirect effect	−0.280 (–0.136)	(–0.372, −0.188)
HrQoL ~ Performance ~ FoF	−0.105 (–0.051)	(–0.182, −0.027)
HrQoL ~ Performance ~ Capacity ~ FoF	−0.068 (–0.034)	(–0.118, −0.017)
HrQoL ~ Disability ~ FoF	−0.007 (–0.004)	(–0.024, 0.010)
HrQoL ~ Disability ~ Performance ~ FoF	−0.034 (–0.017)	(–0.079, 0.011)
HrQoL ~ Disability ~ Performance ~ Capacity ~ FoF	−0.022 (–0.011)	(–0.052, 0.008)
HrQoL ~ Affect ~ FoF	−0.007 (–0.004)	(–0.025, 0.010)
HrQoL ~ Affect ~ Disability ~ FoF	−0.003 (–0.001)	(–0.008, 0.003)
HrQoL ~ Affect ~ Disability ~ Performance ~ FoF	−0.012 (–0.006)	(–0.023, −0.001)
HrQoL ~ Affect ~ Disability ~ Performance ~ Capacity ~ FoF	−0.008 (–0.004)	(–0.015, −0.001)
	**R-squared**	
HrQoL	0.237	
Disability	0.556	
Affect	0.117	
Performance	0.631	
Capacity	0.351	
Activity	0.155	

Notes: Standardized results reported with unstandardized estimates in brackets

Activity = physical activity; Capacity = physical capacity; CI = confidence interval; FoF = fear of falling; HrQoL = health-related quality of life, Performance = physical performance

The direct effect of FoF on HrQoL was small with a standardized path coefficient (β) of -0.05 (95% CI -0.19 to 0.09), suggesting that the association between FoF and HrQoL is mediated by the remaining variables in the model (total indirect effect: β=-0.28, 95% CI -0.37 to -0.19). The strongest separate mediator was physical performance with an indirect effect of β=-0.11 (95% CI -0.18 to -0.03). Additionally considering the pathway from FoF through capacity and physical performance to HrQoL (β=-0.07, 95% CI -0.12 to -0.02), the indirect effect increased to β=-0.17 (95% CI -0.30 to -0.05), indicating that > 50% of the total effect and > 60% of the total indirect effect is explained through mobility limitations (capacity and performance combined). Other indirect effects, e.g. through disability alone (β=-0.01, 95% CI -0.02 to 0.01) or through performance and disability (β=-0.03, 95% CI -0.08 to 0.01), were of lesser importance. Similarly, affect did not seem to be a relevant separate mediator (β=-0.01, 95% CI -0.03 to 0.01). However, the pathways through affect alone, through disability and affect, through physical performance, disability and affect, and through physical capacity, physical performance, disability and affect combined still accounted for 11% (β=-0.03, 95% CI -0.06 to -0.01) of the total indirect effect.

## Discussion

This study aimed to develop a path model explaining the association between FoF and HrQoL in a sample of community-dwelling (pre-)frail older adults. In the final model, the direct effect between FoF and HrQoL was negligible, suggesting that the association can mostly be explained via the other pathways in the model, the most relevant indirect effect going through mobility, mainly physical performance. The model showed very good local and global fit, indicating appropriateness for the data.

### Discussion of the results in the context of existing evidence

The results are in line with previous studies finding that indicators of physical capacity and physical performance mediate the association between FoF and HrQoL [21, 22], but unlike the current study, these found a significant remaining direct effect of FoF on HrQoL. One explanation for this divergence could be the less complex models, considering only a few selected mediating pathways, e.g. only physical performance, disability [21], or leg strength/balance and/or gait speed [22]. The largest proportion of the indirect effect in the current study was also explained through physical capacity and physical performance. However, the remaining pathways, although less relevant individually, together made a considerable contribution to explaining the overall effect.

The standardized coefficients of individual paths in the model were often small and below the level of being considered meaningful [50]. For example, the direct effects between FoF and physical activity and between physical activity and physical capacity were β < 0.2, resulting in the explanatory pathways involving physical activity being close to zero. Despite this, physical activity was left in the model, as model fit worsened considerably when excluding the variable. It is also worth noting that the weak (bivariate) association between FoF or physical capacity and physical activity may be due to the measurement of physical activity in the PromeTheus study. In the German PAQ-50+, several low-intensity activities could be mentioned by the participants. These activities, which are probably barely affected by FoF, made up a large proportion of the overall activity level in the sample [51]. Thus, testing the model with alternative measurements of physical activity (e.g. objectively, sensor-based) is desirable. Overall, the current model provides a more comprehensive picture of the potential pathways that explain the relationship between FoF and HrQoL.

### Interpretation of selected pathways

It was hypothesized that individuals with FoF exhibit lower physical performance, because their physical capacity does not allow it or because their FoF hinders them, independently of their physical capacity. The latter could be explained by avoidance behavior or deliberate activity restriction, which has been found to fully mediate the association between FoF and QoL in nursing home residents [52]. Furthermore, physical performance could also be affected by reasons other than limitations in lower extremity function (which is essentially what the SPPB, used to operationalize physical capacity, measures). FoF-related avoidance behavior was not assessed in PromeTheus and was therefore not included in the model. However, a future extension of the model to include this aspect would enable a differentiation between avoidance-related and actual physical performance limitations.

Contrary to what was expected, there was no relevant independent path through disability despite the strong association between function and disability. However, there was a small effect of the path going from FoF through mobility (physical performance alone or via physical capacity and performance), disability and affect to HrQoL. This suggests that by promoting physical capacity and physical performance, disability can also be positively influenced, which in turn translates into a more desirable level of affect and ultimately better HrQoL.

This implies that measures to improve or maintain physical capacity are the most important interventions to reduce the impact of FoF on HrQoL, particularly because of the feedback loop to physical capacity. The overall direction of our model from FoF to HrQoL was based on our research aim to examine the effect of FoF on HrQoL. This was in line with qualitative evidence [53] and is also the basis for improving HrQoL through geriatric interventions by addressing FoF. Therefore, we prioritized including the pathway from FoF to physical capacity. In addition, there may be an effect of physical capacity on FoF, as has been found for postural instability [54] and often theorized [12, 55, 56]. However, it was methodologically not possible to include this reverse pathway, so only the more practically relevant direction from FoF to physical capacity was included in our model. We therefore emphasize that future research should include a bidirectional pathway whenever possible. Given the major direct and indirect impact of FoF on physical performance, people with FoF should be equipped with strategies on how to safely perform daily tasks despite their FoF, e.g. through training programs that explicitly target a transfer of exercises to everyday tasks or integrate the training/exercises into everyday tasks. For example, the Lifestyle-integrated Functional Exercise (LiFE) program fulfils these criteria and has been shown to improve HrQoL as well as physical capacity, physical performance, and physical activity [57]. Based on the relative weakness of pathways going through disability, interventions which solely focus on adapting to limitations in physical capacity and physical performance might be less effective to mitigate the impact on the HrQoL of persons with FoF.

### Limitations and further research directions

This study has several limitations that suggest directions for future research. First, FoF was assessed by the Short FES-I, which in fact asks about concern of falling in different activities. It would be interesting to examine whether the path model still holds true when FoF is measured by alternative instruments (e.g. a single question about fear of falling or the original 16-item FES-I [58]). Second, due to the cross-sectional design of this study, temporality cannot be used to support an assumption of causality. In particular, a model with an inverted structure, i.e. with pathways leading from HrQoL to FoF, would have exactly the same fit. Moreover, alternative models assuming different causal directions are theoretically conceivable (e.g. FoF may not only impact on physical capacity and physical performance, but the association may be bidirectional [59]). Third, measures used in structural equation models (of which path analysis is a subset) should have good content validity, score reliability, and construct validity [47, 60], which was not comprehensively investigated for all instruments used in the model. Future studies could furthermore fully exploit the capabilities of structural equation modelling by using a measurement model with latent constructs described by multiple indicators. In the present study, a measurement model was not used due to the limited number of indicators available per construct. Fourth, the model was fitted based on a relatively small sample of (pre-)frail older adults from a randomized controlled trial with specific characteristics determined by the eligibility criteria of the trial, which limits the generalizability of the findings. A sample size of 5 to 20 times the number of parameters to be estimated is a commonly recommended [47] but also debated rule of thumb for structural equation models [61]. Depending on the threshold applied, the sample size might be too small for the corrected model. Consequently, the model should be verified on an alternative and larger sample to improve confidence in the model [48].

## Conclusions

The study suggests that the association between FoF and HrQoL can be explained by a number of explanatory pathways, leaving only a negligible direct effect of FoF on HrQoL. Most of the indirect effect was explained by mobility, mainly physical performance, indicating that people with FoF should be equipped with strategies to safely perform everyday tasks despite their FoF. The remaining pathways (e.g. through disability or affect) were less relevant individually, but together contributed considerably to the total indirect effect. Future studies may verify the model and the assumed causal directions using alternative samples and/or longitudinal data.

## Electronic supplementary material

Below is the link to the electronic supplementary material.

Supplementary Material 1

## Data Availability

The datasets generated and/or analyzed during the current study are not publicly available due to ethical and confidentiality concerns but are available from the corresponding author upon reasonable request.
