# Supplementary material for: Path model explaining the association between fear of falling and health-related quality of life in (pre-)frail older adults
Source: BMC Geriatr. 2025 Feb 7;25:87. doi: 10.1186/s12877-025-05718-x (PMC11806603; doi:10.1186/s12877-025-05718-x)
Supplement: Supplementary file 1 — Supplementary Material 1 [file 12877_2025_5718_MOESM1_ESM.docx]

# Supplemental material

Table S 1 Fitted covariance matrix of the raw model

|  | HrQoL | Disability | Affect | Performance | Capacity | Activity | FoF | Age | Education | Falls | Sex |
| --- | --- | --- | --- | --- | --- | --- | --- | --- | --- | --- | --- |
| HrQoL | 4.721 |  |  |  |  |  |  |  |  |  |  |
| Disability | 1.233 | 1.938 |  |  |  |  |  |  |  |  |  |
| Affect | 11.789 | 9.060 | 403.986 |  |  |  |  |  |  |  |  |
| Performance | 1.431 | 1.543 | 7.781 | 2.306 |  |  |  |  |  |  |  |
| Capacity | 1.821 | 2.015 | 9.838 | 2.955 | 7.124 |  |  |  |  |  |  |
| Activity | 1.359 | 1.485 | 5.876 | 2.003 | 3.260 | 17.206 |  |  |  |  |  |
| FoF | -3.095 | -3.049 | -17.209 | -4.111 | -5.867 | -3.123 | 19.166 |  |  |  |  |
| Age | -.271 | -1.581 | -1.499 | -1.872 | -3.778 | -.811 | 1.525 | 34.572 |  |  |  |
| Education | -.356 | .353 | 5.542 | 1.729 | 4.047 | -.249 | -1.127 | -8.348 | 98.521 |  |  |
| Falls | -1.009 | -.977 | -.274 | -.884 | -1.591 | -1.904 | 2.088 | -.597 | -.924 | 23.339 |  |
| Sex | .312 | .682 | -9.468 | -1.215 | -.712 | 6.961 | -3.147 | .179 | -16.307 | -.167 | 78.028 |
| Activity=physical activity; Capacity=physical capacity; Education=Years of education; FoF=fear of falling; HrQoL=health-related quality of life, Performance=physical performance. | | | | | | | | | | | |

Table S 2 Fitted covariance matrix of the corrected model

|  | HrQoL | Disability | Affect | Performance | Capacity | Activity | FoF | Age | Education | Falls | Sex |
| --- | --- | --- | --- | --- | --- | --- | --- | --- | --- | --- | --- |
| HrQoL | 4.721 |  |  |  |  |  |  |  |  |  |  |
| Disability | 1.233 | 1.938 |  |  |  |  |  |  |  |  |  |
| Affect | 11.789 | 9.060 | 403.986 |  |  |  |  |  |  |  |  |
| Performance | 1.431 | 1.543 | 7.781 | 2.306 |  |  |  |  |  |  |  |
| Capacity | 1.821 | 2.015 | 9.838 | 2.955 | 7.124 |  |  |  |  |  |  |
| Activity | 1.359 | 1.485 | 5.876 | 2.003 | 3.260 | 17.206 |  |  |  |  |  |
| FoF | -3.095 | -3.049 | -17.209 | -4.111 | -5.867 | -3.123 | 19.166 |  |  |  |  |
| Age | -.271 | -1.581 | -1.499 | -1.872 | -3.778 | -.811 | 1.525 | 34.572 |  |  |  |
| Education | -.356 | .353 | 5.542 | 1.729 | 4.047 | -.249 | -1.127 | -8.348 | 98.521 |  |  |
| Falls | -1.009 | -.977 | -.274 | -.884 | -1.591 | -1.904 | 2.088 | -.597 | -.924 | 23.339 |  |
| Sex | .312 | .682 | -9.468 | -1.215 | -.712 | 6.961 | -3.147 | .179 | -16.307 | -.167 | 78.028 |

Table S 3 Path coefficients of the raw model

|  | Path coefficient | 95% CI |
| --- | --- | --- |
| **Direct effects** |  |  |
| HrQoL~FoF | -.073 (-.036) | (-.202, .056) |
| HrQoL~Performance | .247 (.354) | (.065, .429) |
| HrQoL~Disability | .141 (.220) | (-.030, .312) |
| HrQoL~Affect | .148 (.016) | (.040, .257) |
| Disability~FoF | -.074 (-.024) | (-.172, .024) |
| Disability~Performance | .691 (.635) | (.605, .777) |
| Affect~FoF | -.045 (-.206) | (-.165, .075) |
| Affect~Disability | .301 (4.353) | (.173, .429) |
| Performance~FoF | -.345 (-.119) | (-.422, -.268) |
| Performance~Capacity | .544 (.309) | (.468, .621) |
| Capacity~FoF | -.485 (-.296) | (-.563, -.407) |
| Capacity~Activity | .096 (.062) | (-.011, .204) |
| Activity~FoF | .003 (.003) | (-.136, .141) |
| Activity~Performance | .286 (.784) | (.117, .456) |
| **Selected indirect effects/paths** |  |  |
| Total indirect effect | -.262 (-.128) | (-.345, -.179) |
| HrQoL~Performance~FoF | -.085 (-.042) | (-.151, -.020) |
| HrQoL~Performance~Capacity~FoF | -.065 (-.032) | (-.116, -.014) |
| HrQoL~Disability~FoF | -.010 (-.005) | (-.029, .008) |
| HrQoL~Disability~Performance~FoF | -.034 (-.017) | (-.076, .009) |
| HrQoL~Disability~Performance~Capacity~FoF | -.026 (-.013) | (-.058, .007) |
| HrQoL~Affect~FoF | -.007 (-.003) | (-.024, .011) |
| HrQoL~Affect~Disability~FoF | -.003 (-.002) | (-.009, .002) |
| HrQoL~Affect~Disability~Performance~FoF | -.011 (-.005) | (-.020, -.001) |
| HrQoL~Affect~Disability~Performance~Capacity~FoF | -.008 (-.004) | (-.016, .000) |
|  | R-squared |  |
| HrQoL | .228 |  |
| Disability | .546 |  |
| Affect | .107 |  |
| Performance | .616 |  |
| Capacity | .283 |  |
| Activity | .108 |  |
| Notes: Standardized results reported with unstandardized estimates in brackets.  Activity=physical activity; Capacity=physical capacity; CI=confidence interval; FoF=fear of falling; HrQoL=health-related quality of life, Performance=physical performance | | |
